# Occurrence of classes I and II integrons in *Enterobacteriaceae* collected from Zagazig University Hospitals, Egypt

**DOI:** 10.3389/fmicb.2015.00601

**Published:** 2015-06-23

**Authors:** Mai M. Malek, Fatma A. Amer, Ayman A. Allam, Rehab H. El-Sokkary, Tarek Gheith, Mohamed A. Arafa

**Affiliations:** ^1^Medical Microbiology and Immunology Department, Faculty of Medicine, Zagazig UniversityCairo, Egypt; ^2^Pediatrics Department, Faculty of Medicine, Zagazig UniversityZagazig, Egypt

**Keywords:** integron, *Enterobacteriaceae*, MDR, risk factors, sequencing

## Abstract

Integrons are genetic units characterized by the ability to capture and incorporate gene cassettes, thus can contribute to the emergence and transfer of antibiotic resistance. The objectives of this study were: (1) to investigate the presence and distribution of class I and class II integrons and the characteristics of the gene cassettes they carry in *Enterobacteriaceae* isolated from nosocomial infections at Zagzig University Hospital in Egypt, (2) to determine their impact on resistance, and (3) to identify risk factors for the existence of integrons. Relevant samples and full clinical history were collected from 118 inpatients. Samples were processed; isolated microbes were identified and tested for antibiotic susceptibilities. Integrons were detected by polymerase chain reaction (PCR) and were characterized into class I or II by restriction fragment length polymorphism (RFLP). Integron-positive isolates were subjected to another PCR to detect gene cassette, followed by gene cassette sequencing. Risk factors were analyzed by logistic regression analysis. Seventy-six *Enterobacteriaceae* isolates were recognized, 41 of them (53.9%) were integron-positive; 39 strains carried class I and 2 strains carried class II integrons. Integrons had gene cassettes encoding different combinations and types of resistance determinants. Interestingly, *blaOXA129* gene was found and *ereA* gene was carried on class I integrons. The same determinants were carried within isolates of the same species as well as isolates of different species. The presence of integrons was significantly associated with multidrug resistance (MDR). No risk factors were associated for integron carriage. We conclude that integrons carrying gene cassettes encoding antibiotic resistance are significantly present among *Enterobacteriaceae* causing nosocomial infection in our hospital. Risk factors for acquisition remain to be identified.

## Introduction

The widespread use of antibiotics with the intra- and inter-species transfer of resistant determinants mediated by plasmids, transposons and gene cassettes in integrons have contributed to the rapid transmission of drug resistance in bacterial pathogens, especially among members of the *Enterobacteriaceae* family (Cergole-Novella et al., 2011). Integrons are mobile DNA elements capable of detention and excision of genes, particularly those responsible for antibiotic resistance. Integrons achieve this by site-specific recombination (Hall and Collis, 1995). The different combinations of gene cassettes can contribute to the diverse genetic organization of integrons. There are five different classes of integrons, each encoding a distinct integrase gene (Mazel, 2006). Class I integrons are the most common type present in clinical isolates of the *Enterobacteriaceae* (DeLappe et al., 2003). Class II integrons are associated with the Tn7 transposon, whose transposition activity is directed at specific attachment sites on chromosomes or plasmids (Rodríguez-Minguela et al., 2009). Although the class II integrons share their cassettes pool with the class I integrons, they are distinguished by divergent integrase sequences (Gillings, 2014). Class I integron possess two conserved segments (5′-CS) and (3′-CS) separated by a variable region including the gene cassettes integrated with antibiotic resistant genes. The 5′-CS consists of (*intI*) gene codes for integrase, adjacent recombination site (*att1*) recognized by the integrase and acts as a receptor for gene cassettes, and the promoter (P) which controls the transcription of integrated resistance markers, as these genes do not have their own promoters (Mazel, 2006). The 3′-CS usually includes truncated *qacE* (*qacED1*) and *sul1* genes that confer resistance to quaternary ammonium compounds and sulfonamides, respectively (Paulsen et al., 1993). Gene cassettes typically comprise of a recombination site (attC) and a single-promoter-less gene, most of which encode antibiotic resistance factors (Partridge et al., 2009). Class I integron has been identified as the primary source of antimicrobial resistance genes and are suspected to serve as reservoirs and exchange platforms of resistant genes in a variety of Gram-negative bacteria (Ke et al., 2011).

During the last months, there were complaints from clinicians about the emergence and dissemination of MDR in the intensive care unit (ICU), the orthopedic unit, the neonatology unit and the chest unit. This resistance causes treatment failure, morbidity and sometimes mortality. Preliminary investigations pointed out the possibility of integrons (among other mechanisms of resistance). To the best of our knowledge, little is known about the integrons and their associated gene cassettes in *Enterobacteriaceae* isolates in our hospital specifically, in Egypt generally. In order to provide helpful base-line information for further comparison with follow up studies, we thus conducted the current work. The objectives were: (1) to investigate the presence and distribution of class I and class II integrons and the characteristics of the gene cassettes they carry in *Enterobacteriaceae* isolated from nosocomial infection cases at Zagzig University Hospital, (2) to determine their impact on resistance, and (3) to identify risk factors of the existence of integrons.

## Materials and methods

### Subjects

Over a period of 23 months (May 2012–March 2014), 118 in-patients were enrolled from different clinical departments at Zagazig University Hospital. Patients were included only if they are suspected to have hospital-acquired infection (Horan et al., 2008) and the laboratory results reported Gram-negative bacilli from the infection sites. Clinical units selected were those reported to the Infection Control Unit as having increased incidence of infections caused by resistant-*Enterobacteriaceae*. The demographic and clinical data was collected from all patients.

### Ethical consideration

An informed written consent was taken from each individual after explaining the nature of investigation as well as the purpose of the study in accordance with the ethical standards of the responsible Regional Committee. Participants' data is confidential. Institutional approval was obtained from the Institutional Review Board (IRB) committee.

### Study design

Observational cross sectional study.

### Clinical samples

Clinical samples were collected by systematic random sampling. The origin and type of samples are shown in Table 1. Samples were collected and processed using standard Microbiologic procedures (Collee and Marr, 1996; Raul and Melvin, 2001; Cheesbrough, 2004; Forbes et al., 2007); isolated microbes were identified by colonial characteristics, Gram stain and conventional biochemical tests and confirmed by the API 20E Identification System (bioMerieux, France).

**Table 1:** **Origin and type of the strains included in the study**.

**Origin**	**No. and types of samples**					**Enterobactericae isolates**	
	**Endotracheal aspiration**	**Urine**	**Swabs from pus**	**Blood**	**Total**		
ICU	8	21	11	12	52	42	(80.8%)
Orthopedic unit	–	9	4	–	13	8	(61.5%)
Neonatology unit	–	–	–	10	10	6	(60%)
Chest unit	5	4	4	2	15	8	(53.3%)
Surgery unit	–	17	11	–	28	12	(42.9%)
Total	13	51	30	24	118	76	(64.4%)

### Antibiotic susceptibility

Antimicrobial susceptibility profiles of the isolates were determined using the disk diffusion method according to the standard procedures of the Clinical and Laboratory Standards Institute (CLSI, 2013). *Escherichia coli* (ATCC25922) (Microbiologics, USA) was used as the quality control strain. The following 17 antibiotic discs (Oxoid) were used: amikacin, amoxicillin+clavulanic acid, ampicillin+sulbactam, azithromycin, cefepime, cefixime, cefotaxime, cefoxitin, cefperazone, ceftazidime, ceftriaxone, chloramphenicol, gentamycin, imipenem, levofloxacin, meropenem, and trimethoprim-sulphamethexazole. MDR was defined according to the guidelines of the European Society of Clinical Microbiology and Infectious Diseases (Magiorakos et al., 2012).

### Molecular characterization of isolated *Enterobacteriaceae*

#### Integrons detection

DNA was extracted from overnight cultures grown on MacConkey's agar, using the QIAamp DNA Mini Kit (QIAGEN, USA), according to the manufacturer's instructions. The DNA was quantified following the recommendations of Surzycki (2000).

#### PCR detection of integrons

Integrons were detected by PCR with the degenerate primers designed to hybridize conserved regions of encoded integrase genes *intI1*, *intI2*, hep35 (5′-TGCGGGTYAARGATBTKGATTT-3′), and hep36 (5′-CARCACATGCGTRTARAT-3′) give a PCR product of 491 bp (White et al., 2001).

### RFLP for differentiation of class I and class II integrons

#### Digestion of PCR product

Using *Rsa I* restriction enzyme (White et al., 2001), PCR products were subjected to digestion with *Rsa I* as follow, 10 μl of the amplified gene segment, 2 μl of the l0x buffer supplied, 1 μl of the enzyme and complete reaction with sterile water to attain the final volume of 20 μl, which was incubated for 3 h at 37°C. Then, the total volume was loaded on the agarose gel. After digestion, integrase I gave rise to one fragment of 491 bp, while integrase II gave rise to two fragments of 334 and 157 bp, respectively.

#### Amplification of gene cassettes of class I and class II integrons

Class I integron cassette structures were amplified using hep58 (5′-TCATGGCTTGTTATGACTGT3-3′) and hep59 (5′-GTAGGGCTTATTATGCACGC-3′) which bind 3′-CS and 5′-CS conserved segments, respectively. Class II integron cassette regions were amplified using hep74 (5′-CGGGATCCCGGACGGCATGCACGATTTGTA-3′), which binds to *attI2* and hep51 (5′-GATGCCATCGCAAGTACGAG-3′), which binds to *orfX* situated downstream of the cassette region within Tn*7* (GenBank accession number AJ002782) (White et al., 2001). PCR was performed for 30 cycles, each cycle consisted of 94°C for 30 s, 55°C for 30 s and extension at 72°C for 45 s for amplification of the integrase genes, or 4 min for amplification of the cassette region. Amplification cycles were performed with DNA thermal cycler (Biometra, Germany), as mentioned elsewhere (White et al., 2000). For each batch of PCR reactions, a positive and negative control was included. Positive control was an isolate confirmed as integrase positive by DNA sequencing. PCR products were analyzed in parallel with a DNA MW-marker (Fermentas) by electrophoresis on 2% agarose gel.

### DNA sequencing

Sequencing reactions were performed through using the BigDye Terminator version 3.1 Cycle Sequencing Kit (Applied Biosystems). Each reaction mixture contained 8 μl of the BigDye Terminator ready reaction mixture and 3.2 pmol of primer in a 20 μl reaction mixture. The PCR products of inserted gene cassettes within class I intgrons were sequenced using primers hep58, 5′-TCATGGCTTGTTATGACTGT-3′ and hep59, 5′-GTAGGGCTTATTATGCACGC-3′. The PCR program for all sequencing reactions included initial denaturation at 96°C for 1 min, followed by 25 cycles of denaturation for 30 s at 96°C, primer annealing for 5 s at 55°C, and extension for 4 min at 60°C according to the BigDye Terminator v3.1 Cycle Sequencing Kit Protocol Manual. The resulting sequences were identified by partial nucleotide sequencing and compared with the sequences in the GenBank database of the National Center for Biotechnology Information via the BLAST network service (http://blast.ncbi.nlm.nih.gov/Blast.cgi) (Li et al., 2013). MEGABLAST model of BLAST program was used. The best Blast Hits on our Query nucleotide sequence was selected based on the highest identity in the gene bank database.

### Statistical analysis

Collected data were computerized and statistically analyzed using SPSS program (Statistical Package for Social Science) version 18.0. Qualitative data were represented as frequencies and relative percentages. Chi square test was used to calculate difference between qualitative variables. Quantitative data were expressed as mean ± SD (Standard deviation). Multivariate logistic regression analysis was used to illuminate the interrelation within and between significant predictors for specific variable. The level of significance for all statistical tests was determined. The threshold of significance is fixed at 5% level (*P*-value); *P* > 0.05 indicates non-significant results, *P*-value of <0.05 indicates significant results.

## Results

Seventy-six *Enterobacteriaceae* isolates were studied. They were recovered from different clinical samples collected from 118 in-patients admitted to different departments at Zagazig University Hospitals (Table 1). The highest rate of isolating *Enterobacteriaceae* was from ICU (42 out of 76).

**Figure 1:**
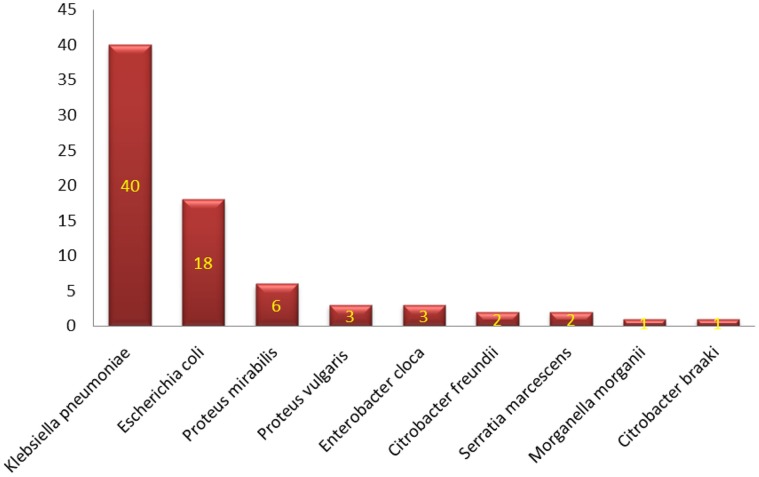
**Distribution of different species of Enterobacteriacae**.

**Figure 2:**
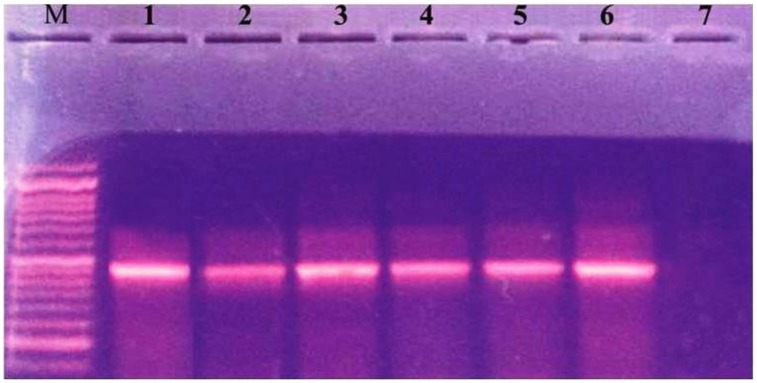
**Ethidium bromide-stained agrose gel showing +ve isolates for integrase gene**. Lane M: molecular size marker (100–1000). Lane 1: positive control. Lanes 2–6: 491 (bp PCR product from positive strains). Lane 7: negative control.

**Figure 3:**
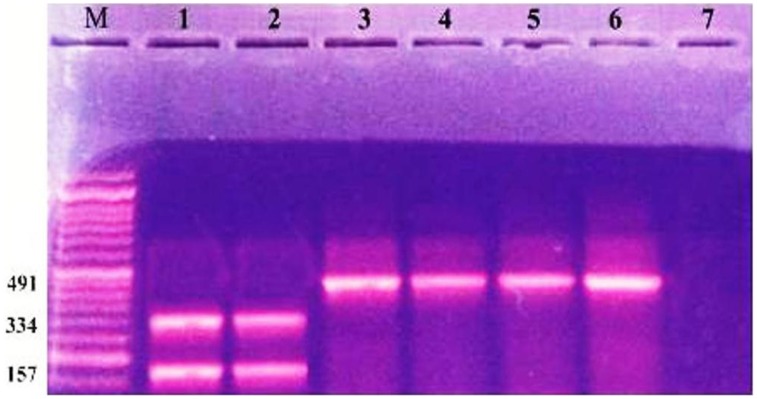
**Ethidium bromide stained agrose gel showing +ve class I and class II integron with RFLP by restriction enzyme digestion**. Lane M: molecular size marker (100–1000). Lanes 1, 2: 2 segments (bands) 334, 157 bp, respectively. Lanes 3–6: 491 (bp PCR product class 1 positive strain). Lane 7: negative control.

Characterization of different species of *Enterobacteriaceae* is shown in Figure 1. *Klebsiella pneumoniae* was the most common organism isolated (50.6%). Integrons were identified in 41 (53.9%) out of the 76 strains studied (Table 2). Thirty-nine (39) carried class I integron and 2 strains carried class II integron Figures 2, 3.

**Table 2:** **Detection of class I and class II integrons by PCR–RFLP**.

***Enterobacteriaceae* isolates**		**Class I**	**Class II**	**Total**
**Organism**	**No.**	**No. (%)**	**No. (%)**	**No. (%)**
*Klebsiella pneumonia*	40	17 (42.5)	1 (2.5)	18 (45)
*Escherichia coli*	18	14 (77.8)	1 (5.5)	15 (83.3)
*Enterobacter cloaca*	3	1 (33.3)		2 (66.6)
*Citrobacter freundii*	2	1 (50)		1 (50)
*Serratia marcescens*	2	1 (50)		1 (50)
*Morganella morgani*	1	0 (0.0)		0 (0.0)
*Proteus mirabialis*	6	3 (50)		3 (50)
*Proteus vulgaris*	3	1 (33.3)		1 (33.3)
*Citrobacter breakie*	1	0 (0.0)		0 (0.0)
Total	76	39 (51.32)	2 (2.6)	41 (53.9)

Sequencing of PCR product of gene cassettes carried on class I integron revealed different combination of gene cassettes encoding the different types of resistance determinants. Interestingly, *blaOXA*129 gene was found and *ereA* gene was detected on class I integrons. The same determinants were carried within isolates of the same species as well as isolates of different species (Table 3).

**Table 3:** **Occurrence and characterization of gene cassettes carried by of class I integrons**.

**Bacterial species**	**Gene cassette(s)**	**No.**
*K. pneumonia*	*dfrA14-arr2-cmiA 5*	2
	*bla_OXA-1_-aac A6-dfrA 5*	2
	*dfrA5-aph 31a*	2
	*dfrA5-TEM_−1_*	1
	*OXA 129-dfrA5-aacA4*	1
	*dfrA*	3
	*aacC1-aadA1*	1
	*aadA1-dfrA1*	3
	*aadA2*	2
	*aadA-qac-e*	2
*E. coli*	*aadB*	1
	*aadA1-dfrA1*	2
	*AadA*	3
	*aac6 II*-	2
	*ere A2-dfr A5*	2
	Not determined	2
*P. mirabilis*	*ere A2-dfr A5*	2
	Not determined	1
*E. cloaca*	*dfr A5*	1
	*DfrA1-aadA2*	1
*S. marcescens*	*blaP1-aadB*	1
*C. freundii*	*dfrA-aadA5-qac-e*	1
*P. vulgaris*	*OXA 129-dfrA5-accA4*	1

*dfr, Dihydrofolate reductases; arr, rifampicin resistance; cmi, chloramphenicol resistance protein; OXA, oxasillinase; aac, aminoglycoside acetyltransferases; aph, 3-O-phosphotransferase; aad, aminoglycoside adenylyltransferases B; qac, quaternary ammonium compound; TEM, beta lactamase gene; bla P, beta lactamase gene*.

Isolates harboring integrons were more likely to be resistant to amikacin, cefepime, ceftazidime, ceftriaxone, chloramphenicol, gentamycin, levofloxacin, and trimethoprim-sulphamethexazole than those without integrons (Table 4). All *Enterobacteriaceae* isolates were sensitive to both imipenem and meropenem; therefore, carbapenems group was excluded from statistical analysis. Fifty-one isolates were found to be MD-resistant (Table 5). A significant statistical association was found between MDR and the presence of integrons (Table 6).

**Table 4:** **Association between antibiotic resistance and integron positivity**.

**Antibiotic**	**Resistant**		**Integron +ve**		***P***
	**No.**	**(%)**	**No.**	**(%)**	
Amikacin	22	31.5	18	81.8	0.0164
Amoxicillin+clavulanic	76	100	40	52.6	0.779
Ampicillin+sulbactam	66	86.8	35	35.03	0.7634
Azithromycin	64	84.2	35	54.7	0.646
Cefepime	47	61.8	33	70.3	0.0302
Cefixime	34	44.7	18	52.9	0.731
Cefotaxime	45	59.2	25	55.6	0.586
Cefoxitin	25	32.8	11	44.0	0.806
Cefperazone	51	67.1	36	70.6	0.169
Ceftazidime	44	57.8	29	65.9	0.0163
Ceftriaxone	53	69.7	39	73.6	0.006
Chloramphenicol	20	26.3	16	80.0	0.0293
Gentamycin	29	28.9	23	79.3	0.0108
Levofloxacin	11	14.4	10	90.9	0.0273
Trimethoprim-sulphamethexazole	48	63.1	39	81.3	0.001

*Level of significance: P < 0.05 indicates significant results*.

**Table 5:** Resistance profile of 51 MDR^*^ isolate.

**Bacterial isolates**	**Antimicrobial categories**	**MDR(51)**	
		**Integron+ve (33)**	**Integron −ve (18)**
*Klebsiella pneumoniae*	Aminoglycosides, extended-spectrum cephalosporins; folate pathway inhibitors, penicillins+β-lactamase inhibitors, chloramphenicol, cephamycins	5	1
	Extended-spectrum cephalosporins; folate pathway inhibitors, penicillins+β-lactamase inhibitors, cephamycins	8	4
	Extended-spectrum cephalosporins; folate pathway inhibitors, penicillins+b-lactamase, quinolones, aminoglycosides	3	0
	Penicillins+b-lactamase inhibitors, chloramphenicol, cephamycins	1	1
	Extended-spectrum cephalosporins, penicillins+β-lactamase inhibitors, chloramphenicol, cephamycins	0	4
*Escherichia coli*	Penicillins+β-lactamase inhibitors, chloramphenicol, cephamycins	3	2
	Extended-spectrum cephalosporins; folate pathway inhibitors, penicillins+β-lactamase inhibitors, quinolones	2	1
	Aminoglycosides, extended-spectrum cephalosporins; folate pathway inhibitors, penicillins+β-lactamase inhibitors, Chloramphenicol, Cephamycins	2	1
*Proteus mirabilis*	Extended-spectrum cephalosporins; folate pathway inhibitors, penicillins+β-lactamase inhibitors	1	2
	Folate pathway inhibitors, penicillins+β-lactamase inhibitors, cephamycins	1	0
*Proteus vulgaris*	Aminoglycosides, extended-spectrum cephalosporins; folate pathway inhibitors, penicillins+β-lactamase inhibitors, chloramphenicol, cephamycins	1	0
	Aminoglycosides; folate pathway inhibitors, penicillins+β-lactamase inhibitors, chloramphenicol, cephamycins, quinolones	1	1
*Serratia marcescens*	Aminoglycosides, extended-spectrum cephalosporins; folate pathway inhibitors, penicillins+β-lactamase inhibitors, chloramphenicol, cephamycins	1	0
	Extended-spectrum cephalosporins; folate pathway inhibitors, penicillins+β-lactamase inhibitors, cephamycins, quinolones	1	0
*Citrobacter freundii*	Aminoglycosides, Extended-spectrum cephalosporins; folate pathway inhibitors, penicillins+β-lactamase inhibitors, cephamycins	1	0
*Enterobacter cloaca*	Folate pathway inhibitors, penicillins+β-lactamase inhibitors, cephamycins, quinolones, chloramphenicol	1	1
*Morganella morgani*	Aminoglycosides, Extended-spectrum cephalosporins; folate pathway inhibitors, penicillins+β-lactamase inhibitors, chloramphenicol cephamycins	1	0

* *Categories used in this table to identify MDR are those recommended by the European Society of clinical Microbiology and Infectious Diseases (Magiorakos et al., 2012): 1, aminoglycosides: gentamicin, amikacin; 2, carbapenems: imipenem, meropenem; 3, extended-spectrum cephalosporins (third and fourth generation cephalosporins); cefotaxime or ceftriaxone, ceftazidime, cefepime; 4, cephamycins: cefoxitin; 5, quinolones: ciprofloxacin, levofloxacin; 6, folate pathway inhibitors: trimethoprim-sulphamethoxazole; 7, Penicillins + β-lactamase inhibitors: amoxicillin-clavulanic, ampicillin-sulbactam; 8, Phenicols: chloramphenicol*.

**Table 6:** **Relation between integron presence and MDR among *Enterobactericae* isolates**.

**Integron**	**Antibiotic**		***χ*^2^**	***P*-value**
	MDR^*^ No. (%)	**Other isolates No. (%)**		
Integron +ve (*N* = 41)	33 (64.7)	9 (36)	4.491	0.017^*^
Integron −ve (*N* = 35)	18 (35.3)	16 (64)		
Total	51 (100)	25 (100)		

χ^2^, Chi square test;

* *level of significans (p < 0.05)*.

Preliminary analysis using Chi square test revealed admission to ICU, trauma and hospitalization more than 7 days to be significantly correlated with integron positivity (Table 7). However, logistic regression analysis revealed no significant association (Table 8).

**Table 7:** **Association between risk factors and presence of integron (Chi square test)**.

**Risk factor**	**No. of cases**	**Integron +ve**		**Integron −ve**		***P*-value**
		**No**	**%**	**No**	**%**	
Trauma	55	38	69.1	17	30.9	0.029^*^
Hospitalization more than 7 days	20	17	85	3	15	0.011^*^
ICU admission	34	29	85.3	5	14.7	0.002^*^
Ventilator	10	8	80	2	20	0.235
Urinary catheter	28	7	25	21	75	0.357
Central venous catheter	5	4	80	1	20	0.586

* *Chi square test*.

**Table 8:** **Logistic regression analysis for significant predictors of the presence of integron among the studied groups**.

**Variable**	***B***	**S.E.**	**Wald**	**Sig.**	**OR**
Trauma	22.02	7.26	0.02	0.98	3.64 (0.07–5.15)
Hospitalization more than 7 days	20.17	8.27	0.04	0.96	5.73 (1.02–7.45)
ICU admission	42.03	1.10	0.03	0.90	0.01 (0.00–0.06)

*B, Regression coefficients; S.E., standard error around the coefficient for the constant; Wald, Wald chi-square test statistic; Sig. P-value for Wald test; OR, odds ratio*.

## Discussion

Members of the family *Enterobacteriaceae* are frequently identified as etiological agents of nosocomial infections (Holt et al., 1994; Obeng-Nkrumah et al., 2013). Hospitalized patients, especially those admitted to ICUs, are at high risk (Archibald et al., 1997; El-Bialy and Abu-Zeid, 2009; Elsharkawy et al., 2013). This could explain the highest rate of isolating *Enterobacteriaceae* from the ICU encountered in the current work (Table 1), which support findings of others (Obeng-Nkrumah et al., 2013). In our study *Klebsiella pneumoniae* was the most commonly isolated organism, followed by *Escherichia coli* (Figure 1). This agrees with Abdel-Hady et al. (2008), Obeng-Nkrumah et al. (2013) and Defife et al. (2009), but disagrees with Asencio et al. (2014). The differences can be due to variations in study population, sample size, the presence of an epidemic as well as dissimilarities in antibiotic use.

The rate of integron-positive *Enterobacteriaceae* identified in this work is 53.9% compared to what was previously reported (White et al., 2001; Kor et al., 2013). We found the highest percentage of integrons in *Escherichia coli* (83.3%) which correlate with the report of Essen-Zandbergen et al. (2007), but not with Daikos et al. (2007) (Table 2). This could be attributed to diverse geographical distribution. The predominance of class I integrons among our isolates agree with many workers (Chang et al., 2000; White et al., 2001; Kor et al., 2013).

PCR product of cassette regions of class I integron were sequenced. The cassette regions of three class I integrons could not be amplified, possibly due to the lack of a 3′-conserved segment or due to presence of early stop codon (Ramírez et al., 2010). It is worth mentioning that the number and combination of genes found in gene cassettes of our isolate are different from that reported by many other researchers (White et al., 2001; Leverstein-van Hall et al., 2002; Kang et al., 2005), which prove the effect of geographic difference on integron distribution (Yu et al., 2004). Class II integons were excluded from sequencing analysis due to the very small sample size which limits the power of any conclusions (Table 3).

In this study, class I integrons harbored different cassette arrays conferring resistance to nearly every major class of antibiotics, with the remarkable exception of the quinolones (Daikos et al., 2007; Essen-Zandbergen et al., 2007). The most common types were those conferring resistance to trimethoprim antibiotic (*dfr*) a finding in agreement with Daikos et al. (2007) and Essen-Zandbergen et al. (2007). The high prevalence of *dfr* gene cassettes may be due to the wide use of trimethoprim as a first line therapy for the treatment of urinary tract infections, a clinical condition that is very common in both hospital and community settings. Another common types of cassette carried by class I integrons were those conferring resistance to streptomycin and spectinomycin (White et al., 2001). Aminoglycoside resistance genes (*aadB, aac*, and aph) encoding resistance to aminoglycosides other than streptomycin and spectinomycin (gentamicin, kanamycin, and amikacin) were found in 11 of our isolates. The selective pressure exerted by aminoglycosides intensively used in our hospitals might account for this finding (Table 3). Resistance to beta-lactam antibiotic was represented in this work by four cassettes (*bla OXA 129-OXA 1-TEM-blaP1*). To the best of our knowledge, *bla OXA 129* is detected for the first time in Egypt. Till date, only two reports are available on the presence of *OXA 129* in Gram-negative bacteria. The first was in 2008 from Brazil, from *Salmonella enterica* subsp. *enterica* serovar *Bredeney* porcine isolates (Michael et al., 2008), and the second was from China, in 2010 from *Pseudomonas aeruginosa* (Liu et al., 2010). The *blaOXA-1* gene was found in two of our isolates. All the genes for the *OXA-1*-like beta-lactamases were identified in the form of gene cassettes inserted into class I integrons (Naas and Nordmann, 1999; Aubert et al., 2001; Dubois et al., 2003; Poirel et al., 2004). Other cassettes detected were those for erythromycin (*ereA2*), and for rifampicin resistance. These two gene cassettes were identified relatively more recently than the other cassettes (Jones et al., 1997; Tribuddharat and Fennewald, 1999; Leverstein-van Hall et al., 2002). The *ereA* gene cassette carries its own promoter and is propagated by a class II integron (Biskri and Mazel, 2003). Recently, a cassette carrying a gene and showing about 90% identity with *ereA* was identified in a class I multi-resistant integron (MRI) (Chang et al., 2000; Peters et al., 2001; Thungapathra et al., 2002; Plante et al., 2003). In this study, the occurrence of *ereA* gene carried on class I integron on four isolates (Table 3) is reported in Egypt for the first time. Chloramphenicol resistance protein (exporter), (*cmiA5*), were detected in two isolates. This gene is responsible for a less prevalent non-enzymatic mechanism of chloramphenicol resistance observed principally in Gram-negative bacteria (Bissonnette et al., 1991). The extremely low occurrence of chloramphenicol resistance, through a less prevalent mechanism presented by gene cassettes of our isolates, can be explained by the fact that chloramphenicol is no more prescribed in hospitals for the fear of its complications. Cassette (*qac-e*) which encodes for quaternary ammonium compounds resistance was detected in three of our isolates. In *Enterobacteriaceae*, the *qac* genes have been most regularly found together with genes coding for resistance to chloramphenicol, aminoglycosides, beta-lactams, sulphonamides and trimethoprim (Poirel et al., 2000; Riaño et al., 2006; Espedido et al., 2008; Zhao et al., 2012). In our work, *qac-e* has been found together with genes encoding resistance to aminoglycosides, and sulphonamides (Table 3). The correlation between the *qac* genes and macrolide inactivation genes was reported in *Aeromonas hydrophila* (Poole et al., 2006) and in microflora from a wastewater treatment unit (Szczepanowski et al., 2004). Hence, the use of several cationic biocides may also be accountable for the selection of bacteria resistant to antimicrobials (Russell, 2000).

It is worth mentioning that not only particular genes are shared across species but also some gene combinations. In the current work, *dfrA5* was present across *Klebsiella pneumoniae, Escherichia coli, Proteus mirabilis, Enterobacter cloaca*, and *Proteus vulgaris*. Gene combination, *aadA1-dfrA1*, was identified in *Klebsiella pneumonia* and *Escherichia coli*; the combination comprised of *ereA2-dfrA5* was detected in *Escherichia coli* and *Proteus mirabilis* (Table 3). We also observed the presence of more than one isolate of *Klebsiella pneumoniae, Escherichia coli* and *Proteus mirabilis* carrying a particular integron (Table 3). Since all these gene cassettes are carried by class I integron which contains integrase 1 (conserved sequence) (White et al., 2001), the transfer of the integrons by both intra- and inter-species is assumed. Integron spread may be achieved through the cross-transmission of integron-carrying clones from one patient to the other, an action known to be facilitated among *Enterobacteriaceae* in hospital settings (Leverstein-van Hall et al., 2002). This is particularly important in the background of poor compliance with infection control in our hospital.

The phenotypic resistance to a specific drug was observed in all isolates carrying the corresponding gene cassette; this coincides with Leverstein-van Hall et al. (2002); Barlow et al. (2004); Kor et al. (2013), and Li et al. (2013). However, it is also evident that integron-carrying organisms had reduced susceptibility not only to antimicrobial agents for which the respective gene cassettes were contained in but also to other classes of agents for which no or very little number of genes are contained within the integrons. In the current work, this applies to the third generation cephalosporins, chloramphenicol, and quinolones. Thus, we support the finding of previous investigators (White et al., 2001; Leverstein-van Hall et al., 2002; Essen-Zandbergen et al., 2007) regarding cephalosporins. Remarkably, not all the resistance profile of the isolates could be explained by the expression of the gene cassettes found within the integrons. Apparently a considerable number of antibiotic resistance genes are located outside the integrons either on chromosomes or plasmids.

More than half of our isolates were MD-resistant (Tables 5, 6) which has also been concluded by other workers (Elsharkawy et al., 2013; Ali et al., 2014). Just as reported by Partridge et al. (2009), class I integrons are significantly associated with MDR.

Logistic regression analysis for risk factors of the presence of integron among the studied group revealed statistically non-significant results (Table 8), which correlate with findings of Daikos et al. (2007). It may be due to small sample size. Hence, further investigation on a larger sample size is recommended.

We conclude that integrons carrying gene cassettes encoding antibiotic resistance are significantly present among *Enterobacteriaceae* causing nosocomial infection in our hospital. We also report for the first time in Egypt the detection of *blaOXA129* and the carriage of *ereA* gene on class I integrons. These existences have important implications. Unless the use of antimicrobials in our hospital is rationalized, the emergence and the dissemination of these genes as well as other encoding resistance to more antibiotics will be evident in the future. Our study has renewed interest in the use of chloramphenicol since it is an effective and a cheap antimicrobial. In low-income countries, the use of chloramphenicol should be encouraged. The use of several cationic biocides should also be appropriate, since inappropriate usage may account for the selection of bacteria resistant to antimicrobials. A significant relationship was found between integron and MD-resistant phenotype. It is to be emphasized that the continuing use of antibiotics will drive the numbers of MDR to swell. The risk factors of integron carriage need to be identified. Our study is providing the base-line information that can be helpful in further monitoring and for evaluation.

### Conflict of interest statement

The authors declare that the research was conducted in the absence of any commercial or financial relationships that could be construed as a potential conflict of interest.
